# A Large Gastrointestinal Stromal Tumor under the Disguise of a Gastric Diverticulum: Report of a Case and Review of the Literature

**DOI:** 10.70352/scrj.cr.24-0133

**Published:** 2025-04-02

**Authors:** Vasileios I. Lagopoulos, Eleni Gigi, Stavros Savvakis, Maria Sidiropoulou, Ioannis Gkoutziotis, Panagiotis-Konstantinos Emfietzis

**Affiliations:** 1 3rd Surgical Department, AHEPA University Hospital of Thessaloniki, School of Medicine, Aristotle University of Thessaloniki, Thessaloniki, Greece; 2 2nd Internal Medicine Department, Hippokration General Hospital of Thessaloniki Aristotle University of Thessaloniki, Thessaloniki, Greece; 3 Computed Tomography Department, Hippokration General Hospital of Thessaloniki, Thessaloniki, Greece; 4 5th Department of Surgery, Hippokration General Hospital of Thessaloniki, Aristotle University of Thessaloniki, Thessaloniki, Greece

**Keywords:** gastrointestinal stromal tumor, gastric diverticulum, differential diagnosis, gastrointestinal hemorrhage, case report

## Abstract

**INTRODUCTION:**

Gastrointestinal stromal tumors (GISTs) are a relatively rare clinical entity. They usually appear as solid masses in numerous locations throughout the gastrointestinal tract, varying in size and typically exhibiting extraluminal expansion along with a range of nonspecific symptoms. The exophytic growth pattern of these tumors may occasionally complicate the differential diagnosis from other medical conditions with similar clinical and imaging findings.

**CASE PRESENTATION:**

We describe a case of a 46-year-old male patient who presented to the emergency department with symptoms of upper gastrointestinal tract hemorrhage. Initial endoscopic findings suggested a large gastric diverticulum. Surprisingly, further investigation with computed tomography and a second endoscopy with biopsy sampling revealed that the stomach wall outpouching was actually a disguised, oversized gastric GIST. The patient underwent a posterior wall sleeve gastrectomy en bloc with the mass, the spleen, and the tail of the pancreas and recovered uneventfully. Daily administration of imatinib as adjuvant therapy was included in the treatment plan. No recurrence was observed even up to the 4-year follow-up period.

**CONCLUSIONS:**

GISTs are uncommon tumors with the ability to masquerade as gastrointestinal tract diverticula, causing diagnostic confusion. Nevertheless, high clinical suspicion combined with a thorough clinical and imaging evaluation can ultimately lead to the correct diagnosis and an appropriate treatment plan.

## Abbreviations

CPAP
: continuous positive airway pressure
CT
: computed tomography
FFP
: fresh frozen plasma
GI
: gastrointestinal
GIST
: gastrointestinal stromal tumor
HPF
: high-power fields
RBC
: red blood cell
TK
: tyrosine kinase

## INTRODUCTION

Gastrointestinal stromal tumors (GISTs) are relatively rare clinical entities with an incidence of approximately 10–14.5 new cases per million inhabitants per year in European countries.^1)^ They were recognized as a distinct group of mesenchymal tumors in the 1980s.^2)^ Previously, GISTs were mostly classified as leiomyosarcomas until Mazur and Clack^3)^ noticed that some of these tumors are found positive for the S-100 protein—a marker for cells derived from neuroectoderm—and raised the question of whether they originate from the myenteric nerve plexus. It is now widely accepted that they derive from the interstitial cells of Cajal, which act as pacemakers for gut motility, although some researchers claim that they may originate from mesenchymal stem cells.^4)^ Anatomically, about two-thirds of GISTs are found in the stomach, with most of the remainder located in the small and large bowel.^5)^ Clinical findings are related to both the size and the anatomic location of the tumors. Small GISTs may be asymptomatic and identified incidentally, while larger tumors usually cause symptoms ranging from a mild abdominal discomfort to gastrointestinal (GI) tract hemorrhage, or even acute abdomen in the case of a rupture.^6,7)^ Due to their exophytic growth pattern outside the GI tract lumen, diagnosis through endoscopy can be very challenging. Indeed, we report a case of a patient with upper GI hemorrhage and an initial incorrect endoscopic diagnosis of a gastric diverticulum. During further workup, the interesting endoscopic finding ultimately proved to be a large gastric GIST with central necrosis, and the patient was managed accordingly. Additionally, a brief review of the literature was conducted to identify similar clinical scenarios, with the aim of informing clinical practitioners about the potential of GISTs to masquerade as GI tract diverticula.

## CASE PRESENTATION

**Fig. 1:**
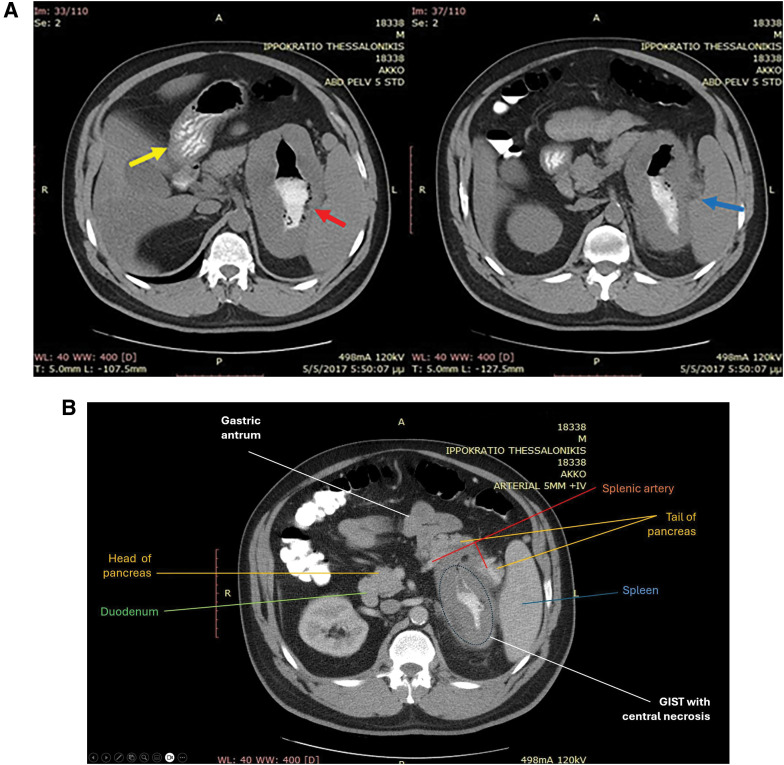
(**A**) Abdominal computed tomography scan of the patient. Two diverging lumens are filled with gastrografin: 1 lumen shows visible gastric folds (yellow arrow), while the other contains undigested material (red arrow). The mass appears to be adhered to the tail of the pancreas and the splenic hilum (blue arrow). (**B**) Anatomical relationships of the tumor with the surrounding structures, as seen on the abdominal computed tomography scan. Possible infiltration or strangulation of the tail of the pancreas. A “blurring” is described between the tumor and the splenic artery. The tumor also appears to be adhered to the splenic hilum. GIST, gastrointestinal stromal tumor

A 46-year-old male patient was admitted to the emergency room of Hippokration General Hospital of Thessaloniki, complaining of multiple episodes of melena over the last 48 hours, accompanied by mild epigastric pain. He recalled that the onset of pain occurred about a week ago, for which he was taking ibuprofen without professional medical advice. Upon clinical examination, the patient was hemodynamically stable, with no findings other than the referred epigastric pain and a rectal examination that was positive for blood. His medical history indicated that he was being treated for dyslipidemia and used a continuous positive airway pressure device during sleep for sleep apnea. The patient had not undergone any surgical procedure in the past, and his family history was clear of GI conditions. Laboratory tests revealed hypochromic microcytic anemia, with hemoglobin at 9.6 mg/dL and no other abnormal values. Initial endoscopic investigation reported the presence of a giant diverticulum with an ulcerated neck along the lesser curvature of the stomach, filled with undigested content. Biopsies taken from the neck of the diverticulum were negative for malignancy, showing a mixture of normal and necrotized mucosa. A computed tomography (CT) scan was then performed to evaluate the size and relations of the diverticulum and to explore treatment options. The imaging findings (**Figs. 1A** and **1B**) showed a 13 × 14 × 15 cm mass arising from the posterior wall of the cardia, expanding leftward and caudally toward the greater curvature. It came into close contact with the spleen and compressed the tail of pancreas, as well as the left kidney and left adrenal gland. Both the surgical and radiology teams agreed that these findings were more consistent with a GIST or another malignancy, and less indicative of a diverticulum. Therefore, a second endoscopy was performed, which revealed that the interior of the outpouching lacked normal mucosa, and new biopsies were taken. Preoperative pathological examination of the biopsy specimen included hematoxylin and eosin (HE) staining and immunohistochemical analysis. HE staining revealed spindle-cell morphology, while immunohistochemical staining was positive for c-kit, dog1, and CD34, and negative for desmin and smooth muscle actin (SMA). All the above confirmed the actual presence of a stromal tumor rather than a gastric diverticulum.

**Fig. 2:**
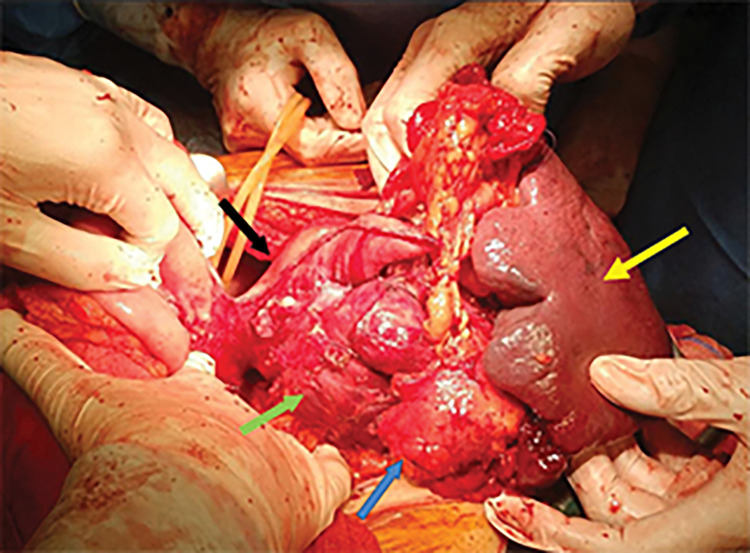
Intraoperative picture. Sleeve gastrectomy, distal pancreatectomy, and splenectomy were performed for en bloc removal of giant GIST (green arrow) of the posterior gastric wall. The tail of the pancreas (blue arrow) has been dissected, and the gastric fundus (black arrow) and the spleen (yellow arrow) are fully mobilized from their ligaments. The dissection of the splenic artery, right after its origin from the coeliac trunk, was the last step of the procedure. GIST, gastrointestinal stromal tumor

**Fig. 3:**
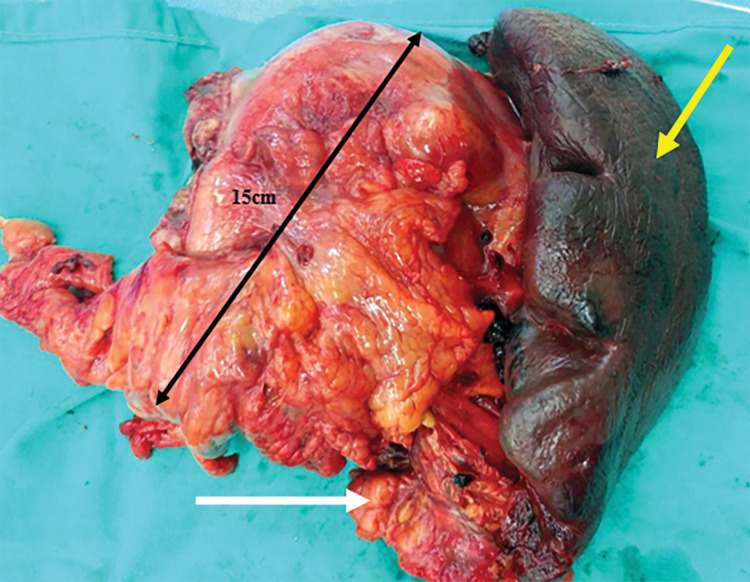
The resected specimen. En bloc removal of the tumor (15 cm in diameter, black arrow) along with the distal pancreas (white arrow) and the spleen (yellow arrow).

Throughout the course of diagnostic testing, the patient continued to have melena, necessitating the administration of 7 units of red blood cells (RBCs) and 5 units of fresh frozen plasma (FFP) to maintain hemodynamic stability. After evaluation, the multidisciplinary oncologic council suggested surgery as the optimal treatment choice. A median supraumbilical incision was made to enter the peritoneal cavity, and no signs of distant or peritoneal metastasis were encountered. The lesser sac was accessed via an incision in the left gastrocolic ligament, and the tumor appeared to extend from the posterior wall of the gastric fundus to the splenic hilum and the tail of the pancreas. The splenic vessels, as well as the serosa of the spleen, seemed strongly attached to the periphery of the tumor, giving the impression of infiltration (**Fig. 2**). Blunt dissection was attempted, but was unsuccessful. Based on both the radiological and surgical findings, en bloc resection of the tumor, spleen, and tail of the pancreas was considered the optimal approach. The gastric fundus was mobilized, and the splenophrenic ligament was divided. The splenic artery was dissected in the middle of the upper edge of the pancreas. The tail of the pancreas was carefully dissected from the splenic vein posteriorly and divided using a linear stapler. The splenic vein was dissected and divided peripherally to the inferior mesenteric vein. The spleen was released from its lateral ligaments and retracted centrally. The greater omentum was divided at the level of the gastric antrum, and a sleeve gastrectomy was performed, extending cephalad to the left of the gastroesophageal junction. The specimen was resected, and hemostasis was achieved. A continuous suture with Vicryl 2/0 (Atlas Medical, Athens, Greece) was used to reinforce the suture line of the gastric remnant. Finally, a latex tube was placed for drainage of the left upper quadrant, and closure of the abdominal wall was performed in its anatomic layers. The patient was transfused with 2 units of RBC and 1 unit of FFP intraoperatively. The surgical time was recorded as 115 minutes and the blood loss was estimated at 150 mL. The final pathology report showed a red-brownish stromal tumor, positive for c-kit, dog1, and CD34, and negative for desmin and SMA, with central necrosis, measuring 15 cm in maximum diameter. The tumor was attached to but not infiltrating the spleen and the tail of pancreas. The surgical margins, as well the 10 lymph nodes retrieved, were also negative, and the tumor was consequently characterized as T_4_N_0_, according to the UICC 8th edition TNM staging system^8)^ (**Fig. 3**). The mitotic count was not higher than 5/50 high-power fields (HPF). According to Miettinen and Lasota classification,^5)^ this gastric GIST (>10 cm, mitotic count ≤5/50 HPF) falls into the moderate-risk category for recurrence. Due to its gastric origin and low mitotic activity, the estimated recurrence risk is approximately 12% over 10 years. The patient received adjuvant treatment with imatinib, 400 mg daily, in accordance with the proposal by the multidisciplinary oncologic council. Follow-up evaluations included an abdominal ultrasound, laboratory testing, and clinical examination by an oncologist every 6 months, while a CT scan was performed annually. No recurrence was observed even up to the 4-year follow-up period.

All procedures performed in this study were in accordance with the ethical standards of the institutional and/or national research committee(s) and with the Declaration of Helsinki (as revised in 2013). Written informed consent was obtained from the patient for the publication of this case report and the accompanying images. A copy of the written consent is available for review by the editorial office of this journal.

## DISCUSSION

The stomach is the most common primary location of GISTs, accounting for an estimated 55.6% of GI tract cases.^9)^ To the best of our knowledge, this is the first published case of a gastric GIST that masquerades as a gastric diverticulum. Gastric diverticula, on the other hand, represent the most unusual form of GI tract diverticulum and a very rare anatomical abnormality overall, with an estimated prevalence of 0.01%–0.11%.^10)^ There are cases in the literature which prove that these 2 medical entities (GISTs and GI diverticula) can sometimes be confused during the initial diagnostic procedures.

A search of the literature pinpointed 2 separate cases of duodenal GISTs that mimicked large duodenal diverticula.^11,12)^ Okasha et al.^11)^ proposed that the rapid growth of the tumor, coupled with the insufficiency of its internal blood supply, causes central necrotic cavitation and liquefaction. Consequently, a fistulization between the tumor cavity and the lumen occurs, allowing air or fluid to enter the cavity. During the first endoscopy of our patient, the presence of air and fluid in an outpouching of the stomach wall misleadingly created the false impression of a large diverticulum.

In the first case of duodenal GIST, the patient complained of a prolonged vague abdominal discomfort, while in the second case, the patient presented with upper GI bleeding.^11,12)^ Our patient exhibited multiple episodes of melena, along with mild referred epigastric pain. The aforementioned symptoms are obviously not a pathognomonic feature for GISTs, as these tumors can manifest with a variety of unspecific clinical symptoms depending on their anatomical location.^12)^ They may even be completely asymptomatic and discovered incidentally.^13)^ A retrospective study examining 800 GIST patients from the Mannheim GIST registry revealed that the most common clinical presentation of gastric GISTs is the upper GI bleeding, which may manifest as acute hematemesis, melena, or even as chronic microcytic anemia.^14)^ The current case aligns with these findings, as the patient presented with melena, while chronic microcytic anemia was additionally found in the laboratory tests. The bleeding may be attributed to ulceration of the mucosal surface by the growing submucosal tumor.^14)^ A prospective study from Italy demonstrated that GI bleeding and abdominal pain are the most frequent symptoms of gastric GISTs, with a palpable epigastric mass being the third most frequent finding.^15)^

Similar cases of diagnostic confusion between GISTs and large diverticula have also been reported in anatomical areas other than the stomach and duodenum. Two case reports involving jejunal and sigmoid colon GISTs presented the same dilemma, while another case described an ileal GIST that was initially mistaken for a Meckel’s diverticulum. All 3 of these cases exhibited more significant clinical manifestations compared to gastric and duodenal GISTs, including perforation and subsequent peritonitis, which required emergency laparotomy.^16–18)^

Clinical imaging plays an indispensable role in the diagnosis and decision-making for GISTs.^19)^ In the current case, an abdominal CT scan with gastrografin was deemed necessary to examine the actual size and anatomical relations of the suspected diverticulum. This imaging gave us the opportunity to question the initial diagnosis of gastric diverticulum due to its impressive size and exophytic growth pattern outside the stomach. Another “red flag” supporting the diagnosis of a gastric GIST rather than a diverticulum was the fact that the scan showed 2 divergent lumens filled with gastrografin, with gastric folds visible only in the left one. The second lumen resembled the necrotic cavity of the tumor, filled with undigested content, with no view of gastric mucosa. Lastly, the anatomical relations of the outpouching with the surrounding tissues were not suggestive of a diverticulum. The close contact with the spleen and the compression of the tail of the pancreas are generally not rare phenomena when encountering large gastric GISTs, as these tumors tend to invade into the gastrohepatic ligament, gastrosplenic ligament, and lesser sac.^20)^

The gold standard of treatment for localized disease is surgical removal via a desired R0 resection.^21)^ That is usually achieved for gastric GIST by a wedge resection en bloc with the tumor and any adhered structures, with partial or even total gastrectomy as secondary options, depending on tumor’s size and relations.^21)^ Lymph node resection is not recommended as GISTs do not metastasize via the lymphatic route.^22)^ Extreme care should be taken to avoid tumor rupture, which significantly increases the possibility of intraabdominal seeding.^22)^ In accordance with the 2023 GEIS Guidelines for GISTs,^23)^ we performed a posterior wall sleeve gastrectomy en bloc with the mass, the spleen, and tail of the pancreas. Even though complete surgical removal is achievable for most localized GISTs, about 40% of patients experience metastatic relapse.^24)^ Two phase III randomized clinical trials evaluated the effectiveness of adjuvant imatinib in curbing disease relapse and enhancing overall survival in GIST patients, most of whom had gastric GISTs.^25,26)^ The ACOSOG Z9001 and SSG XVIII/AIO studies demonstrated improved relapse-free survival at 1 and 3 years, respectively, with daily adjuvant imatinib at a dose of 400 mg.^25,26)^

## CONCLUSIONS

GISTs are rare neoplasms that can be completely asymptomatic or present with a variety of symptoms. The diagnosis of these tumors is usually not problematic as they have characteristic endoscopic and imaging findings. However, large tumors with central necrosis might mimic other exophytic structures, such as large GI tract diverticula. In such cases, clinical suspicion should remain high, and “red flags” that are more indicative of a GIST rather than a diverticulum should be assessed, as the treatment strategies differ. Complete surgical resection, if achievable, is the cornerstone of therapy, with tyrosine kinase inhibitors as an alternative for metastatic disease or as adjuvant treatment.

## DECLARATIONS

### Funding

The authors received no financial support in association with this case report.

### Authors’ contributions

VIL and IG engaged in primary case management. MS evaluated the computed tomography scan and provided the images.

SS, EG, and P-KE conducted the review of the literature.

All authors met the 4 criteria recommended by the International Committee of Medical Journal Editors (ICMJE); made a substantial contribution to the conception or design of the work, or the acquisition, analysis, or interpretation of data for the work; drafted the manuscript or revised it critically for important intellectual content; approved the final version of the manuscript for publication; and agreed to be held accountable for all aspects of the work.

### Availability of data and materials

The datasets supporting the conclusions of this article are included within the article.

### Ethics approval and consent to participate

Written informed consent was obtained from the patient for the publication of this case report and all accompanying images. The identity of the patient was protected.

### Consent for publication

Consent for the publication of this case report was obtained from the patient.

### Competing interests

The authors declare that they have no competing interests.

## References

[1] Xu L Ma Y Wang S Incidence of gastrointestinal stromal tumor in Chinese urban population: a national population-based study. Cancer Med 2021; 10: 737–44. 33320439 10.1002/cam4.3644 PMC7877389

[2] Joensuu H. Gastrointestinal stromal tumor (GIST). Ann Oncol 2006; 17(Suppl 10): x280–6. 17018739 10.1093/annonc/mdl274

[3] Mazur MT Clark HB. Gastric stromal tumors. Reappraisal of histogenesis. Am J Surg Pathol 1983; 7: 507–19. 6625048 10.1097/00000478-198309000-00001

[4] Kara T Serinsoz E Arpaci RB Contribution of DOG1 expression to the diagnosis of gastrointestinal stromal tumors. Pathol Res Pract 2013; 209: 413–7. 23722018 10.1016/j.prp.2013.04.005

[5] Miettinen M Lasota J. Gastrointestinal stromal tumors: pathology and prognosis at different sites. Semin Diagn Pathol 2006; 23: 70–83. 17193820 10.1053/j.semdp.2006.09.001

[6] Giljača V Grohovac D Kovač D Gastrointestinal stromal tumors characteristics in Croatian Northern Adriatic region. Hepatogastroenterology 2012; 59: 2512–5. 23178617 10.5754/hge10436

[7] Sorour MA Kassem MI Ghazal Ael-H Gastrointestinal stromal tumors (GIST) related emergencies. Int J Surg 2014; 12: 269–80. 24530605 10.1016/j.ijsu.2014.02.004

[8] Brierley JD Gospodarowicz MK Wittekind C. The TNM classification of malignant tumours. 8. Oxford: Wiley Blackwell; 2017.

[9] Søreide K Sandvik OM Søreide JA Global epidemiology of gastrointestinal stromal tumours (GIST): A systematic review of population-based cohort studies. Cancer Epidemiol 2016; 40: 39–46. 26618334 10.1016/j.canep.2015.10.031

[10] Shah J Patel K Sunkara T Gastric diverticulum: a comprehensive review. Inflamm Intest Dis 2019; 3: 161–6. 31111031 10.1159/000495463 PMC6501548

[11] Okasha HH Amin HM Al-Shazli M A duodenal gastrointestinal stromal tumor with a large central area of fluid and gas due to fistulization into the duodenal lumen, mimicking a large duodenal diverticulum. Endosc Ultrasound 2015; 4: 253–6. 26374586 10.4103/2303-9027.163018 PMC4568640

[12] Gupta N Schirmer BD Mishra R Malignant GIST masquerading as a bleeding duodenal diverticulum. Endoscopy 2007; 39(Suppl 1): E142–3. 17611891 10.1055/s-2007-966245

[13] Parab TM DeRogatis MJ Boaz AM Gastrointestinal stromal tumors: a comprehensive review. J Gastrointest Oncol 2019; 10: 144–54. 30788170 10.21037/jgo.2018.08.20 PMC6351301

[14] Menge F Jakob J Kasper B Clinical presentation of gastrointestinal stromal tumors. Visc Med 2018; 34: 335–40. 30498699 10.1159/000494303 PMC6257088

[15] Caterino S Lorenzon L Petrucciani N Gastrointestinal stromal tumors: correlation between symptoms at presentation, tumor location and prognostic factors in 47 consecutive patients. World J Surg Oncol 2011; 9: 13. 21284869 10.1186/1477-7819-9-13 PMC3039617

[16] Arata R Nakahara H Urushihara T A case of a diverticulum-like giant jejunal gastrointestinal stromal tumour presenting with intraperitoneal peritonitis due to rupture. Int J Surg Case Rep 2020; 69: 68–71. 32283516 10.1016/j.ijscr.2020.03.017 PMC7154945

[17] Shintaku Y Asano Y Watanabe T A case of planar-type GIST of the sigmoid colon showing diverticular structure with perforation. World J Surg Oncol 2020; 18: 125. 32527279 10.1186/s12957-020-01906-8 PMC7291680

[18] Omerza CR Bouman AK Bulinski PP. A rare case of a gastrointestinal stromal tumor (GIST) presenting as a perforated Meckel’s diverticulum. J Gastrointest Cancer 2017; 48: 76–9. 26820464 10.1007/s12029-016-9803-y

[19] Lau S Tam KF Kam CK Imaging of gastrointestinal stromal tumour (GIST). Clin Radiol 2004; 59: 487–98. 15145718 10.1016/j.crad.2003.10.018

[20] Chourmouzi D Sinakos E Papalavrentios L Gastrointestinal stromal tumors: a pictorial review. J Gastrointestin Liver Dis 2009; 18: 379–83. 19795038

[21] Bischof DA Kim Y Dodson R Open versus minimally invasive resection of gastric GIST: a multi-institutional analysis of short- and long-term outcomes. Ann Surg Oncol 2014; 21: 2941–8. 24763984 10.1245/s10434-014-3733-3

[22] Nishimura J Nakajima K Omori T Surgical strategy for gastric gastrointestinal stromal tumors: laparoscopic vs. open resection. Surg Endosc 2007; 21: 875–8. 17180273 10.1007/s00464-006-9065-z

[23] Serrano C Martín-Broto J Asencio-Pascual JM 2023 GEIS Guidelines for gastrointestinal stromal tumors. Ther Adv Med Oncol 2023, 15. 10.1177/17588359231192388 PMC10467260 37655207

[24] Joensuu H Vehtari A Riihimäki J Risk of recurrence of gastrointestinal stromal tumour after surgery: an analysis of pooled population-based cohorts. Lancet Oncol 2012; 13: 265–74. 22153892 10.1016/S1470-2045(11)70299-6

[25] Dematteo RP Ballman KV Antonescu CR Adjuvant imatinib mesylate after resection of localised, primary gastrointestinal stromal tumour: a randomised, double-blind, placebo-controlled trial. Lancet 2009; 373: 1097–104. 19303137 10.1016/S0140-6736(09)60500-6 PMC2915459

[26] Joensuu H Eriksson M Sundby HK One vs three years of adjuvant imatinib for operable gastrointestinal stromal tumor: a randomized trial. JAMA 2012; 307: 1265–72. 22453568 10.1001/jama.2012.347

